# Mediating Effect of Social Capital on the Association Between Digital Literacy and Life Satisfaction Among Older Adults in South Korea: Cross-Sectional Study

**DOI:** 10.2196/68163

**Published:** 2025-02-06

**Authors:** Hyein Jung, Hocheol Lee, Eun Woo Nam

**Affiliations:** 1 Department of Health Administration, Yonsei University Graduate School, Wonju, Republic of Korea; 2 Division of Health Administration, College of Software and Digital Healthcare Convergence, Yonsei University, Unit 412, Chang-jo gwan 1 Yonseidae-gil, Wonju, 26493, Republic of Korea, 82 337602413, 82 337629562

**Keywords:** digital literacy, digital access, digital competency, digital utilization, life satisfaction, older adults, social capital, mediating effect, aging

## Abstract

**Background:**

As Korea rapidly transforms into a super-aged society, research indicates that digital literacy among older adults enhances their life satisfaction. Digital literacy refers to the ability to efficiently use digital technologies, encompassing access, competency, and utilization. It reflects the capacity to navigate and benefit from digital environments effectively. Furthermore, social capital positively influences the quality of life, and digital literacy facilitates social capital formation. However, since most studies have only focused on the direct relationship between digital literacy and life satisfaction, research on the mediating role of social capital remains limited.

**Objective:**

To analyze the effect of digital literacy on the life satisfaction of older adults in South Korea and to verify whether social capital acts as a mediating factor in this process.

**Methods:**

This descriptive cross-sectional study used data from the 2023 Report on the Digital Divide—an annual survey conducted by the Korean Ministry of Science and Information and Communications Technology. The study targeted individuals aged 65 years or older. Descriptive statistics, the Pearson correlation analyses, and the 3-step multiple regression analysis proposed by Baron and Kenny were performed. The bootstrap method was employed, and all analyses were conducted using R, version 4.4.1.

**Results:**

The study included 869 participants. Digital literacy had a significant positive effect on their life satisfaction (*β*=0.103; *P*=.008). Social capital was also positively associated with life satisfaction (*β*=0.337; *P*<.001). Mediation analysis showed that digital literacy influenced life satisfaction both directly (*β*=0.103; *P*=.006) and indirectly through social capital (*β*=0.037; *P*=.03). Bootstrapping confirmed the significance of the indirect effect (*β*=0.037, 95% CI 0.005‐0.070; *P*=.03). The total effect of digital literacy on life satisfaction was also significant (*β*=0.140, 95% CI 0.058‐0.230; *P*=.002).

**Conclusions:**

This study analyzed the association between digital literacy, social capital, and life satisfaction among older adults in Korea. We identified that social capital mediates the association between digital literacy and life satisfaction among older adults. These findings indicate that tailored digital literacy programs and support policies that promote social capital formation could help bridge the digital divide and foster social inclusion. These measures would enable older adults to access essential services, reduce social isolation, and enhance health and well-being, ultimately improving the overall quality of life.

## Introduction

South Korea is rapidly approaching a super-aged society, where over 20% of the population is 65 years and older. As of 2024, approximately 9.9 million, or 19.2%, of the total population fall with in this age group. By 2025, this is projected to exceed 20%, officially marking the country’s transition into a super-aged society [1]. This increase in the older adult population has led to a decrease in the proportion of the economically active population. Therefore, the financial and psychological burdens at the individual, family, and national levels are expected to increase due to aging-related diseases and care issues. In 2019, the medical expenses of older adults in Korea accounted for over 40% of the nation’s total medical expenses, and the country recorded the highest elderly poverty rate among the Organisation for Economic Co-operation and Development (OECD) countries [2]. Moreover, the life satisfaction of older adults aged 65 years and over decreased by 2.4% compared to the preceding year, reaching 31.9%, which is lower than the overall average. Life satisfaction also tends to decrease with age [1].

Enhancing the digital technology proficiency of older adults has been proposed as a potential solution to address these challenges [3,4]. Although internet use has grown significantly in recent years, disparities remain, especially among older adults. In South Korea, older adults aged 55 years and above are categorized as one of the 4 major digitally vulnerable groups, alongside people with disabilities, low-income households, farmers, and fishermen. Among these groups, older adults have the lowest level of digital literacy, highlighting a significant digital divide [5]. This digital gap limits access to essential resources and opportunities, hindering efforts to improve their quality of life.

As social systems become increasingly digitized, older adults experience considerable difficulties in managing various daily tasks, such as internet-based purchases, bill payments, and financial transactions. Furthermore, the rapid expansion of telemedicine and digital health care services following the COVID-19 pandemic has significantly increased reliance on digital platforms for health management. This shift has exacerbated challenges for older adults in effectively utilizing these technologies [6,7]. These difficulties are further intensified by resistance to new technologies, psychological barriers, and negative self-perceptions related to aging [4,8,9]. Consequently, these challenges isolate older adults socially and economically and hinder their ability to lead an independent and fulfilling life.

Digital literacy is the ability to efficiently use digital technologies, grounded in fundamental literacy skills such as reading, writing, and arithmetic. It encompasses digital access, digital competency, and digital utilization, enabling individuals to search, evaluate, utilize, share, and create content using digital tools such as the internet and smartphones [10,11].

Research has shown that digital literacy positively impacts the life satisfaction of older adults by enhancing personal development, social connections, and self-esteem [12]. Activities such as searching for financial and health information and engaging in internet-based communication boost self-efficacy, foster positive perceptions of aging, alleviate loneliness and isolation, and improve overall quality of life [13-15]. Domestic research has also demonstrated that the digital literacy level of older adults positively impacts their life satisfaction [16-18]. Moreover, it plays a crucial role in maintaining and expanding relationships with family, friends, and the community, thereby positively influencing the formation of social capital [19,20].

Social capital, which refers to the social networks, norms, and trust that arise from interactions among individuals and groups, serves as a resource that promotes a sense of community and enables individuals to lead fulfilling lives [21,22]. Previous studies have identified social capital as a vital resource that helps alleviate loneliness and enhances life satisfaction, thereby positively influencing the mental well-being of older adults [23-27]. When older adults effectively utilize digital technologies, their social capital is further strengthened, helping them overcome social isolation and enhance their quality of life [19]. Specifically, the enhancement of digital literacy contributes to the growth of social capital, thereby improving emotional stability and life satisfaction [15]. Digital literacy allows older adults to connect with broader social networks, which elevates their overall quality of life.

While several studies have explored the relationship between digital literacy and life satisfaction among older adults and the connection between social capital and quality of life, most have focused only on their direct relationships. Thus, research on how social capital mediates this connection remains limited. This highlights the need to better understand how digital literacy contributes to life satisfaction, considering the potentially mediating role of social capital. Therefore, this study aims to analyze the impact of digital literacy on the life satisfaction of Korean older adults and to verify whether social capital acts as a mediating factor in this process.

## Methods

### Study Design and Aim

This cross-sectional study is aimed at analyzing the mediating effect of social capital on the relationship between digital literacy and life satisfaction among older adults in Korea.

### Data Collection

This study used raw data from the 2023 Report on the Digital Divide—an annual survey conducted by the Korean Ministry of Science and Information and Communications Technology (MSIT). Considering the OECD’s definition of older adults and the age criteria commonly used for pension and social security benefits, this study defines older adults as those aged 65 years and above. Accordingly, response data from this demographic were extracted, processed, and analyzed. The survey employed a structured questionnaire administered through face-to-face interviews. Measures were taken to minimize response bias by standardizing the interview process and providing training for surveyors. The survey period lasted from October to December 2023. For analysis, the data were deidentified in compliance with the Personal Information Protection Act. The sample was selected using a proportional and stratified probability sampling method, accounting for inclusion probability based on region, gender, and age, and estimates of standard error for each stratum. Among the 1239 participants aged 65 years and older, data from 869 individuals were analyzed after excluding responses with missing values (n=370).

### Study Variables

#### Life Satisfaction

The dependent variable in this study, life satisfaction, was measured across 8 domains of daily life: leisure and cultural activities, financial status, social activities, interpersonal relationships, family relationships, work, physical and mental health, and political engagement. These were assessed using a 4-point Likert scale, and the scores were converted to a 100-point scale. A Cronbach α of 0.806 was calculated to assess the internal consistency of the measurement items.

#### Digital Literacy

The independent variable in this study, digital literacy, was evaluated by integrating 3 components: digital access, digital competency, and digital utilization. Digital access was assessed using 4 items, examining whether participants owned digital devices such as desktops, laptops, and mobile phones and whether they had constant internet access (1 for yes, 0 for no). Digital competency was measured using 7 items on personal computer competency and 7 items on mobile device competency, each assessed using a 4-point Likert scale ranging from “Strongly disagree” (1) to “Strongly agree” (4). Digital utilization was assessed based on 50 items categorized by wired or mobile internet usage, the diversity of internet services used, and advanced internet utilization. The diversity of internet service usage included activities such as information and news searches, email, media content, educational content, social networking services, messengers, blogs, internet-based communities, cloud services, daily information, e-commerce, financial transactions, and public service utilization. Advanced internet utilization measured the extent of information production and sharing, networking, social participation, and economic activities. All items were rated on a 4-point Likert scale ranging from “Do not use at all” (1) to “Use frequently” (4). Scores for digital access, digital competency, and digital utilization were calculated on a weighted 100-point scale, as outlined in the 2023 Report on the Digital Divide. A Cronbach α of 0.967 confirmed the internal consistency of these items.

#### Social Capital

The mediating variable, social capital, was measured by assessing the presence of social support networks, such as family and friends, in the internet-based environment for older adults. The survey used a shortened version of the Internet Social Capital Scale (ISCS) developed by Williams [28], modified and adapted by the National Information Society Agency of Korea to evaluate social capital levels. The original ISCS consists of 20 items, with 10 items each for bonding social capital and bridging social capital. Bonding social capital assesses aspects such as emotional support, access to limited resources, and the ability to mobilize solidarity. Bridging social capital evaluates aspects such as an outward-looking perspective, contact with a wide range of people, a sense of belonging to a larger group, and interactions with broader communities. In this study, the shortened scale with 5 items each for bonding and bridging social capital, totaling 10 items, was used. All items were rated on a 4-point Likert scale from “Strongly disagree” (1) to “Strongly agree” (4) and converted to a 100-point scale, with a Cronbach α of 0.863 confirming reliability.

#### Control Variables

The control variables included sociodemographic characteristics such as gender, age, education level, disability status, household type, and monthly income. Age was categorized into 65‐69, 70‐74, 75‐79, and 80 years or older. Education level was classified as lower than elementary school, middle school graduate, high school graduate, and college graduate or higher. Disability status was binary (“yes” or “no”), and household type was classified as either single-person households or households with 2 or more people. Monthly income was divided into 5 quintiles based on income levels.

### Data Analysis

The data were analyzed using the R statistical program, version 4.4.1, and the specific analysis methods are as follows. First, descriptive statistical analyses, including frequency analysis, percentages, means, and SDs, were conducted to understand the participants’ sociodemographic characteristics, digital literacy level, life satisfaction, and social capital. Second, the Pearson correlation analyses were performed to identify the associations between the main variables of the study model: digital literacy, life satisfaction, and social capital. Third, to verify the mediating effect of social capital in the association between digital literacy and life satisfaction, a 3-step multiple regression analysis based on Baron and Kenny’s procedure [29] was conducted. The bootstrap method was applied to test the statistical significance of mediation effects, using a 95% CI.

### Ethical Considerations

This study was conducted in accordance with the Declaration of Helsinki and received review and approval from the Institutional Research Ethics Committee of the Yonsei University Institutional Review Board in Wonju, South Korea (Approval No.1041849‐202410-SB-213-01). All procedures followed the relevant institutional guidelines and regulations, and informed consent was obtained from all participants or their legal guardians before participation. To ensure privacy and confidentiality, the data used in this study underwent anonymization and deidentification processes.

## Results

### Participants Characteristics

A total of 869 participants were included in this study (Table 1). There were marginally more women (n=450, 51.8%) than men (n=419, 48.2%). By age, the largest group included those aged 65‐69 years old (n=367, 42.2%), followed by those aged 70‐74 years old (n=296, 34.1%), 75‐79 years old (n=164, 18.9%), and 80 years and older (n=42, 4.8%). Regarding education level, high school graduates accounted for the largest proportion (n=345, 39.7%), followed by middle school graduates (n=289, 33.3%), those with elementary school education or lower (n=182, 20.9%), and college graduates or higher (n=53, 6.1%). In terms of disability status, most respondents (n=855, 98.4%) did not have a disability, while 1.6% (n=14) reported having a disability. Household type showed that 18.2% (n=158) lived in single-person households, while 81.8% (n=711) lived in households with 2 or more people. Regarding monthly income, the largest group earned ₩2‐₩2.99 million (US $1392.30-$2081.49) (n=233, 26.8%), followed by ₩1‐₩1.99 million (US $696.15-$1385.34) (n=216, 24.9%), ₩4 million or more (US $2784.61) (n=188, 21.6%), ₩3‐₩3.99 million (US $2088.45-$2777.64) (n=152, 17.5%), and less than ₩1 million (US $696.15) (n=80, 9.2%).

**Table 1.** Respondents’ characteristics and degree of study variables.

Characteristic	Participants (n=869)
Gender, n (%)	
Male	419 (48.2)
Female	450 (51.8)
Age (years), n (%)	
65‐69	367 (42.2)
70‐74	296 (34.1)
75‐79	164 (18.9)
≥80	42 (4.8)
Education level, n (%)	
Elementary school or lower	182 (20.9)
Middle school	289 (33.3)
High school	345 (39.7)
College or higher	53 (6.1)
Disability, n (%)	
No	855 (98.4)
Yes	14 (1.6)
Live with someone, n (%)	
No	158 (18.2)
Yes	711 (81.8)
Income, n (%)^a^	
<1 million	80 (9.2)
1‐1.99 million	216 (24.9)
2‐2.99 million	233 (26.8)
3‐3.99 million	152 (17.5)
≥4 million	188 (21.6)
Digital literacy^b^, mean (SD)^c^	56.2 (10.5)
Digital access	62.4 (16.6)
Digital competency	47.2 (16.5)
Digital utilization	62.2 (6.2)
Life satisfaction, mean (SD)^c^	64.9 (11.0)
Social capital, mean (SD)^c^	67.9 (12.0)

a Income reported in Korean won (₩1=US $0.00070) and categorized into ranges.

b Digital literacy: the sum of access, competency, and utilization, with respective weights of 0.2, 0.4, and 0.4.

c All scores presented on a 100-point scale.

The mean digital literacy score was 56.2 (SD 10.5), with the subcomponents showing the following mean scores: 62.4 (SD 16.6) for digital access; 47.2 (SD 16.5) for digital competency; and 62.2 (SD 6.2) for digital utilization. The mean life satisfaction score was 64.9 (SD 11.0), and the mean social capital score was 67.9 (SD 12.0).

### Correlation Analysis Between Life Satisfaction, Digital Literacy, and Social Capital

The results of the Pearson correlation analyses, conducted to examine the correlation between digital literacy, social capital, and life satisfaction, are presented in Table 2. Life satisfaction showed a statistically significant positive correlation with both digital literacy (*r*=0.276, *P*<.001) and social capital (*r*=0.438, *P*<.001), with a stronger correlation observed between life satisfaction and social capital. This indicates that higher levels of digital literacy and social capital are associated with greater life satisfaction. Additionally, digital literacy showed a significant positive correlation with social capital (*r*=0.216, *P*<.001), indicating that as digital literacy increases, social capital tends to increase as well.

**Table 2.** Correlation analysis of life satisfaction, digital literacy, and social capital.

Variables	Life satisfaction	Digital literacy	Social capital
Life satisfaction			
*r*	—^a^	0.276	0.438
*P* value	—	*P*<.001	*P*<.001
Digital literacy			
*r*	0.276	—	0.216
*P* value	*P*<.001	—	*P*<.001
Social capital			
*r*	0.438	0.216	—
*P* value	*P*<.001	*P*<.001	—

a Not applicable.

### The Mediating Effect of Social Capital on the Association Between Digital Literacy and Life Satisfaction

Following the procedure proposed by Baron and Kenny [29], a 3-step multiple regression analysis was conducted to examine the effects of digital literacy on life satisfaction and the mediating role of social capital (Table 3), controlling for gender, age, education level, disability status, and income level.

**Table 3.** The mediating effect of social capital on the association between digital literacy and life satisfaction.^a^

Variables	Model 1^b^		Model 2^c^		Model 3^d^	
	β (SE)	*t* (*P*^e^)	β (SE)	*t* (*P*^e^)	β (SE)	*t* (*P*^e^)
Gender						
Female (reference: male)	1.253 (0.818)	1.531 (.13)	−0.276 (0.736)	−0.375 (.71)	−0.699 (0.684)	−1.022 (.31)
Age (years)						
70‐74 (reference: 65‐69)	−1.324 (0.934)	−1.417 (.16)	−1.342 (0.840)	−1.597 (.11)	−0.896 (0.780)	1.148 (.25)
75‐79 (reference: 65‐69)	0.172 (1.171)	0.147 (.88)	0.138 (1.053)	0.131 (.90)	0.080 (0.977)	0.082 (.93)
≥80 (reference: 65‐69)	−0.477 (1.940)	−0.246 (.81)	−0.287 (1.745)	−0.164 (.87)	−0.1255 (1.619)	−0.078 (.94)
Education level						
Middle school (reference: ≤ elementary school)	3.213 (1.173)	2.738 (.006)	0.745 (1.055)	0.706 (.48)	−0.339 (0.983)	−0.345 (.73)
High school (reference: ≤ elementary school)	4.244 (1.285)	3.302 (.001)	1.686 (1.156)	1.459 (.15)	0.255 (1.079)	0.236 (.81)
≥ College (reference: ≤ elementary school)	5.206 (2.084)	2.497 (.01)	5.399 (1.875)	2.879 (.004)	3.643 (1.745)	2.087 (.04)
Disability						
Yes (reference: no)	1.038 (3.182)	0.326 (.74)	3.403 (2.862)	1.189 (.24)	3.053 (2.655)	1.150 (.25)
Live with someone						
Yes (reference: no)	0.059 (1.224)	0.048 (.96)	−0.363 (1.101)	−0.330 (.74)	−0.383 (1.021)	−0.375 (.71)
Income^f^						
1‐1.99 million (reference:<1 million)	1.643 (1.655)	0.992 (.32)	2.579 (1.489)	1.732 (.08)	2.025 (1.382)	1.465 (.14)
2‐2.99 million (reference:<1 million)	3.676 (1.775)	2.071 (.04)	5.642 (1.596)	3.534 (<.001)	4.402 (1.484)	2.965 (.003)
3‐3.99 million (reference:<1 million)	4.765 (1.939)	2.458 (.01)	6.524 (1.744)	3.741 (<.001)	4.916 (1.623)	3.029 (.003)
≥4 million (reference:<1 million)	5.502 (1.962)	2.805 (.005)	7.371 (1.765)	4.177 (<.001)	5.515 (1.644)	3.354 (<.001)
Digital literacy	0.110 (0.046)	2.364 (.02)	0.140 (0.042)	3.350 (<.001)	0.103 (0.039)	2.647 (.008)
Social capital	—^g^	—	—	—	0.337 (0.029)	11.81 (<.001)

a All models were adjusted for control variables, including gender, age, education level, disability status, household type, and income level.

b Model 1: effect of digital literacy on social capital (constant=54.969; *R*2=0.096; adjusted *R*2=0.082; *F*_852_=6.51; *P*<.001).

c Model 2: effect of digital literacy on life satisfaction (constant=51.755; *R*2=0.133; adjusted *R*2=0.119; *F*_852_=9.38; *P*<.001).

d Model 3: effect of digital literacy and social capital on life satisfaction (constant=33.214; *R*2=0.255; adjusted *R*2=0.242; *F*_851_=19.48; *P*<.001).

e *P* values <.05 are considered significant.

f Income reported in Korean won (₩1=US $0.00070) and categorized into ranges.

g Not applicable.

Model 1 analyzed the effect of digital literacy on social capital. Digital literacy showed a statistically significant positive effect on social capital (*β*=0.110; *P*=.02), indicating that higher levels of digital literacy tend to increase social capital. Moreover, higher education and income levels were positively associated with social capital.

Model 2 examined the effect of digital literacy on life satisfaction. Digital literacy had a statistically significant positive effect on life satisfaction (*β*=0.140; *P*<.001). Higher life satisfaction was observed among those with a college degree or higher (*β*=5.399; *P*=.004) and those with a monthly income of ₩2‐₩2.99 million (*β*=5.642; *P*<.001), ₩3‐₩3.99 million (*β*=6.524; *P*<.001), and ₩4 million or more (*β*=7.371; *P*<.001).

Model 3 analyzed the effects of digital literacy and social capital on life satisfaction. Both digital literacy (*β*=0.103; *P*=.008) and social capital (*β*=0.337; *P*<.001) had statistically significant positive effects on life satisfaction, indicating that both factors contribute to enhancing life satisfaction. Additionally, higher life satisfaction was observed among those with a college degree or higher (*β*=3.643; *P*=.04) and those with a monthly income of ₩2‐₩2.99 million (*β*=4.402; *P*=.003), ₩3‐₩3.99 million (*β*=4.916; *P*=.003), and ₩4 million or more (*β*=5.515; *P*<.001).

The *F* and *P* values for each model (models 1, 2, and 3) were statistically significant (*P*<.001), indicating that the models fit the data well. Notably, model 3 demonstrated the highest explanatory power, with an adjusted *R*^2^ of 0.242, when social capital was included.

**Figure 1.**
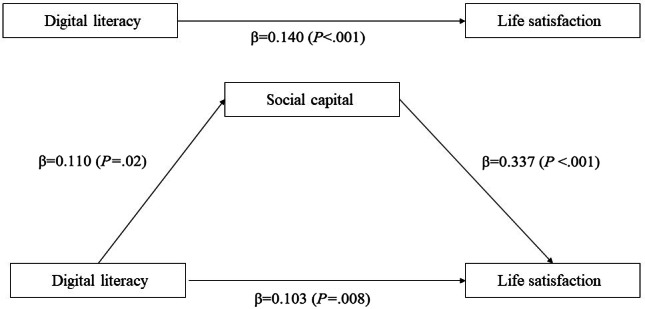
Association between digital literacy, social capital, and life satisfaction.

The regression coefficient of the mediating variable, social capital, was statistically significant, and the direct effect of the independent variable, digital literacy, on the dependent variable, life satisfaction (*β*=0.140), decreased when social capital was included as a mediator (*β*=0.103). This confirms that social capital had a partial mediating effect on the relationship between digital literacy and life satisfaction (Figure 1). Thus, social capital played a key role in enhancing life satisfaction, and digital literacy positively influenced life satisfaction through social capital.

### Verification of the Significance of the Mediating Effect of Social Capital

Table 4 presents the results of the bootstrapping analysis performed to verify the statistical significance of the mediating effect of social capital. First, the direct effect of digital literacy on life satisfaction, without mediation by social capital, was 0.103 (*P*=.006), confirming that digital literacy had a significant direct effect on life satisfaction. The indirect effect, representing the effect of digital literacy on life satisfaction through social capital, was 0.037 (*P*=.03). The total effect, which combines the direct and indirect effects, was 0.140 (*P*=.002), indicating that the overall impact of digital literacy on life satisfaction was statistically significant. Thus, digital literacy was an important factor that positively influenced life satisfaction, both directly and indirectly, through the mediating role of social capital.

**Table 4.** Verification of the significance of the mediating effect of social capital.^a^

Mediation pathway	β (95% CI)	*P* value^b^
Indirect effect		
Digital literacy → Social capital → Life satisfaction	0.037 (0.005-0.070)	.03
Direct effect		
Digital literacy → Life satisfaction	0.103 (0.030-0.180)	.006
Total effect	0.140 (0.058-0.230)	.002

a Results are based on bootstrapping simulations (n=5000).

b *P* values <.05 are considered significant.

## Discussion

### Principal Findings

This study analyzed the effect of digital literacy on life satisfaction among older adults in Korea and investigated the mediating role of social capital in this relationship. The results showed that digital literacy has a positive effect on life satisfaction. These findings align with prior research indicating that higher levels of digital access, competency, and utilization are associated with greater life satisfaction [16,18,30]. Studies on older adults have highlighted the internet’s role as a valuable tool in daily life, supporting interpersonal communication, information searches, task completion, and leisure activities, all of which contribute positively to life satisfaction [31,32]. These findings underscore the internet as a crucial resource that empowers older adults to lead independent and active lives. This study reinforces these findings by demonstrating that a higher level of digital literacy positively influences life satisfaction among older adults in Korea.

The COVID-19 pandemic accelerated the digitalization of public services in Korea, significantly increasing reliance on digital platforms for essential services, including health care, administration, and finance. Although Korea is known for its advanced digital infrastructure, older adults face new challenges in accessing these essential services. This often leads to delays in medical care and limited access to health information, increasing the risk of adverse health outcomes [33]. Moreover, the rapid expansion of financial digitalization has exposed older adults with low digital literacy and limited decision-making abilities to economic risks, such as financial fraud [34]. According to a report comparing OECD countries, Korea’s younger population (aged 25‐34 years) demonstrates digital proficiency levels higher than the OECD average, whereas the older population aged 55‐65 years exhibits proficiency levels below the OECD average. Consequently, Korea is identified as having one of the largest generational digital proficiency gaps among OECD countries [35].

Public and private organizations in Korea have provided digital literacy education for older adults, including training in computer and smartphone usage. However, these programs have predominantly taken the form of one-to-many classroom-style lectures focused on basic skills, which may not sufficiently address the diverse needs of older adults [36]. To better meet these needs, it is recommended to first assess digital literacy levels to determine older adults’ specific digital proficiency levels and then provide tailored training based on these levels. For beginners, training on basic smartphone usage and sending text messages would be appropriate, while intermediate and advanced courses could cover digital health management, internet-based financial education, and the creation of digital photo albums.

Such tailored education can also be observed in international examples. The United Kingdom’s Mi WiFi project (2017‐2018) provided smart devices and digital education to vulnerable groups, including older adults and individuals with disabilities, helping them acquire basic digital skills. Similarly, the United States’ Cyber Seniors project involved young mentors from volunteer organizations visiting older adults in their homes or facilities to provide one-to-one digital education, significantly enhancing their digital competency [37]. The older adult population differs widely in terms of income, education level, and health status. Therefore, digital education programs should be designed to reflect the diverse characteristics and needs of these groups.

In this study, social capital was found to have a positive impact on older adults’ life satisfaction, consistent with previous studies’ findings [38-41]. As social networks shrink and participation opportunities diminish with age, maintaining or acquiring social capital becomes increasingly challenging [42]. Social capital is often divided into 2 dimensions: bonding social capital and bridging social capital [43]. Bonding social capital refers to emotional support, access to resources, and a sense of solidarity formed within close relationships, such as family, friends, and neighbors. When older adults have networks from which they can obtain resources or receive emotional support, their life satisfaction significantly improves. Meanwhile, bridging social capital is formed through interactions with a wider community and diverse individuals, which helps reduce social isolation and contributes to higher life satisfaction among older adults.

These dimensions of social capital are shaped by components such as networks, social participation, norms, and social trust. Among these, social trust in family, friends, neighbors, and institutions was identified as the most important factor affecting life satisfaction in older adults [44]. By strengthening relationships with family and the local community, social trust contributes to psychological stability and enhances the quality of life for older adults. This highlights the need for community-based support environments that consider the unique characteristics of older adults, enabling them to build and maintain social capital. Communities should provide programs and spaces that allow older adults to share their thoughts and experiences, fostering meaningful relationships. Such environments are essential to reinforcing social capital and improving the life satisfaction of older adults.

In this study, social capital showed a partial mediating effect on the relationship between digital literacy and life satisfaction among older adults. This implies that digital literacy not only directly enhances life satisfaction but also indirectly influences it through social capital. These findings are consistent with those of previous research showing that social capital mediates the relationship between digital utilization and life satisfaction among individuals aged 55 years and older [45]. Older adults with higher levels of digital literacy tend to build social relationships and participate in voluntary social activities through internet-based platforms, leading to improvements in their life satisfaction [46-48]. For example, Yoon et al [49] demonstrated that during the COVID-19 pandemic, a pilot program using digital platforms for social prescriptions effectively expanded the social networks of Korean older adults and improved their life satisfaction. South Korean society continues to emphasize family-centered values, with older adults particularly prioritizing their relationships with family members. Maintaining connections with family and friends via social networking services or remote communication positively impacts psychological well-being and overall quality of life [50,51].

Recently, some local governments in Korea have proposed tailored support measures for older adults, categorized based on their digital competency levels and specific needs. For instance, support programs have been planned for groups requiring digital tools for daily life, those seeking social interactions, and those pursuing self-development and cultural enrichment [52]. These programs aim to enable older adults to effectively utilize digital tools, foster social interactions, and mitigate social isolation. Ultimately, it is essential to implement practical support measures that expand social capital in a digital environment and enable older adults to lead more independent and fulfilling lives.

### Limitations and Future Directions

This study has the following limitations. First, as a cross-sectional study, it cannot track changes in causal relationships between variables over time. Future studies should utilize longitudinal data to better understand how relationships between variables change over time. Second, this study focuses on older adults in Korea, which limits the generalizability of the results to other countries or groups with different cultural backgrounds. The relationship between digital literacy and social capital formation may differ across countries, highlighting the need for international comparative studies. Third, most of the study participants (855/869, 98.4%) were not disabled and belonged to the upper-middle-income group, which may restrict the generalizability of the findings. Therefore, the results should be interpreted with caution.

Nevertheless, this study is significant as it used nationwide raw data from the Korean MSIT, analyzing data representative of the older adult population in Korea. It emphasizes the importance of improving digital literacy and expanding social capital to enhance the quality of life of older adults in Korea.

Additionally, future research should explore the distinct impacts of the components of digital literacy—digital access, competency, and utilization—in greater detail. Particularly, examining specific aspects of digital literacy, such as media utilization skills, online safety awareness, and information-seeking behaviors, could help identify their relative effects on life satisfaction among older adults. This analysis would provide critical insights for effectively reducing the digital divide among older adults and designing tailored digital education and support policies to meet their specific needs.

### Conclusion

This study analyzed the associations between digital literacy, social capital, and life satisfaction among older adults in Korea. The results showed significant correlations among digital literacy, social capital, and life satisfaction, with social capital mediating the relationship between digital literacy and life satisfaction. These findings underscore that digital literacy, when combined with social capital, contributes to enhancing the life satisfaction of older adults. Therefore, improving digital literacy and expanding social capital are crucial for promoting the psychological well-being and quality of life of older adults. Furthermore, strengthening digital literacy in the rapidly aging society of Korea can not only improve the quality of life for older adults but also help bridge generational gaps and foster greater social cohesion.

## Abbreviations

OECD: Organisation for Economic Co-operation and Development
MSIT: Ministry of Science and Information and Communications Technology
ISCS: Internet Social Capital Scale
